# On a Case of Prolapsus Uteri, Cured without Operation or the Necessity of Wearing a Pessary

**Published:** 1859-05

**Authors:** E. C. Nourse

**Affiliations:** Brighton


*On a Case of Prolapsus Utcri, cured without Operation or the necessity of Wearing a Pessary.* By E. C. Nourse, Esq., F. R. C. S., Brighton.

A single woman, aged sixty-five, applied to me, in February, 1858, on account of prolapsus uteri, from which she had suffered for three years. She looked haggard and worn, had been losing flesh, strength, and appetite for many months, and complained much of depressed spirits, dragging pain in the loins, and inability for exertion. The womb was generally down, though she could reduce it. I found it hanging between the thighs, about the size of a melon, the mucus membrane dry and glazed, of a reddish brown color, and superficially ulcerated. The orifice of the urethra occupied the usual position in front of the tumor, and the os uteri was seen at the lowest part of it. The womb being replaced, was found very movable and the vagina much relaxed and enlarged.

Considering the success which had attended operations for the cure of prolapsus by partial occlusion of the vagina, I determined to see if I could not apply the principle of these operations in a simpler way. I therefore directed that, now that the womb was replaced, it should never again be allowed to come down, even for a single moment; that a sort of thick pad, or cushion, of a length and breadth sufficient to cover completely the external parts, should be applied, and be kept in its place by a broad and firm T bandage, before she again rose from the recumbent posture; that she should prepare a sufficient number of these pads and T bandages; should always put one on before she rose from her bed in the morning, just as a ruptured person puts on a truss, and should never go about without one; and lastly, that she should introduce every night into the vagina a few grains of tannic acid, made up into a sort of soft pill.

She steadily followed the plan proposed, and at the end of two months had gained strength and flesh, and got rid of all her discomfort. The tannic acid was now used twice a week only, but the pad was directed to be worn constantly. She still continues its use, and reports that the womb has never once come down, and that she has quite recovered her health and spirits, can walk out, and attend to her household duties, quite as well as before she became subject to the affection.—*London Lancet, March,* 1858.

